# Strategic application of multilayer fat grafting in facial rejuvenation: a retrospective study

**DOI:** 10.3389/fsurg.2026.1744865

**Published:** 2026-04-01

**Authors:** Ruomeng Yang, Zhen Song, Jian Wang, Hongwei Liang

**Affiliations:** 1 The Department of Plastic Surgery, The Fifth Clinical Medical College of Henan University of Chinese Medicine (Zhengzhou People’s Hospital), Zhengzhou, China; 2 The Department of Plastic Surgery, Henan Provincial People’s Hospital, Zhengzhou, China

**Keywords:** adipose matrix complex, autologous fat grafting, facial aging, facial rejuvenation, high-density fat, stromal vascular fraction gel

## Abstract

**Backgrounds:**

Facial aging is characterized by complex volumetric changes involving soft tissue atrophy and skeletal remodeling. Autologous fat grafting has evolved as a primary technique, though unpredictable graft retention remains a challenge.

**Objective:**

The study aims to provide the strategic application of differentially processed adipose products by matching material properties to anatomical requirements.

**Methods:**

This retrospective study analyzed 105 patients undergoing facial fat grafting for age-related contour deformities. Adipose products, including high-density fat (HDF), adipose matrix complex (AMC), and stromal vascular fraction gel (SVF-gel), were injected into specific anatomical layers. Outcome assessments included clinical evaluation and standardized photographic documentation, Visual Analog scale, and Global Aesthetic Improvement Scale.

**Results:**

All patients achieved significant aesthetic improvement with no major complications. Early side effects resolved spontaneously within 3 weeks. Sixteen patients required secondary procedures due to partial volume absorption. SVF-gel demonstrated efficacy in periorbital rejuvenation and skin quality enhancement, while HDF and AMC provided stable structural support in deeper facial regions. Patient-reported satisfaction scores were 5.26 ± 1.84 preoperatively and 8.01 ± 1.09 postoperatively. Observer assessments using the Global Aesthetic Improvement Scale (GAIS, −1 to 3) yielded a postoperative score of 1.88 ± 0.65.

**Conclusions:**

The targeted application of processed adipose products based on their biological properties and recipient site requirements enables effective, multilayer facial restoration. This approach addresses both volumetric deficits and skin quality concerns, offering a comprehensive solution for facial rejuvenation.

## Introduction

Facial contour deformities resulting from aging represent a significant concern within cosmetic and reconstructive plastic surgery. The natural aging process typically involves soft tissue loss, skin laxity and ptosis, and craniofacial skeletal alteration (1–3). The deformities involve in critical aesthetic regions such as the temples, malar areas, nasojugal groove, and nasolabial folds, leading to an overall aged appearance that often causes psychological distress for patients (3, 4). Restoring a youthful facial contour has become a primary objective for patients demanding aesthetic improvement.

Autologous fat grafting (AFG) has been successfully applied and modified by Coleman since 1990s (5). AFG emerged as a premier technique for soft tissue augmentation due to its superior biocompatibility, natural results and cost-effectiveness (6, 7). Adipose tissue comprises adipocytes, the stromal vascular fraction (SVF), and the extracellular matrix (ECM) (8, 9). In addition, the SVF includes adipose-derived stem cells (ADSCs), pericytes, and fibroblasts, which have demonstrated potential for tissue regeneration and skin quality enhancement through neovascularization and collagen synthesis (10), offering benefits beyond simple volume replacement. Additionally, compared with alloplastic fillers (11), autologous fat eliminates the risk of allergic reactions, foreign body responses, and can offer a permanent and stable appearance.

A variety of processed adipose tissue products, such as macrofat, microfat, nanofat, and SVF-gel, have been developed for different clinical applications (12). For instance, macrofat, with its abundant structural components, is typically used for volume restoration and structural support. Nanofat, however, has shown significant potential for enhancing tissue regeneration (12–14). Despite several advantages, the widespread adoption of fat grafting is constrained by unpredictable graft retention rates, which remain the most significant challenge in achieving consistent outcomes. Variability in results can be attributed to multiple technical factors including harvesting methods, processing techniques, and injection protocols. These inconsistencies highlight the need for clinical studies to establish standardized protocols that can improve outcomes of facial fat grafting.

To enhance the understanding of the comprehensive application of multiple adipose-derived products, when used in combination across different tissue layers, this study provides a detailed retrospective analysis of patients undergoing autologous fat transfer for facial rejuvenation. The findings may contribute to optimized treatment strategies for achieving consistent and sustained aesthetic outcomes in facial rejuvenation procedures.

## Methods

### Study population and data resource

A retrospective study of 105 patients who received facial fat grafting between September 2022 and February 2025 was conducted. Inclusion criteria were as follows: (1) Age between 18 and 50 years. (2) Presence of facial contour depressions resulting from aging. (3) Willingness to participate in the clinical study and provision of signed informed consent. Exclusion criteria were as follows: (1) Presence of acute facial inflammation. (2) Females during menstruation, pregnancy, or lactation. (3) Individuals with psychological disease. Following approval granted by the Medical Ethics Committee of The Fifth Clinical Medical College of Henan University of Chinese Medicine (Zhengzhou People's Hospital). The study was carried out in adherence to the principles outlined in the Declaration of Helsinki.

### Surgical technique

The inner thigh and lower abdomen were selected as donor sites for fat harvesting. Preoperative markings of both the donor and recipient sites were performed with the patient in standing position. The patient was placed in the supine position. Local tumescent anesthesia was administered in the donor site, and tumescent solution was prepared by combining 1,000 mL of normal saline, 10 mL of 2% lidocaine, and 1 mL of 0.1% epinephrine. Surgery commenced after allowing a 10-minute interval for adequate infiltration of the tumescent solution. Adipose tissue was harvested under low manual negative pressure using a 20 mL syringe connected to a 2.5 mm blunt-tip cannula. A systematic fan-shaped technique was utilized to ensure consistent and uniform extraction.

Based on previously published literature, adipose tissue was processed (6). The harvested adipose tissue was initially processed to excise fibrous components, which were blot-dried on cotton pads and meticulously minced to generate the adipose matrix component (AMC) (Supplementary Video 1). The processed adipose tissue underwent 5 min of vertical standing for gravity separation, followed by decantation of the infranatant tumescent fluid. The processed adipose tissue was then centrifuged at 1,200 g for 3 min, consequently removing the lower infranatant and upper oil layer, yielding high-density fat (HDF) from the retained bottom third (Supplementary Video 2). The remaining upper two-thirds of the fat tissue was mechanically emulsified using two 20-mL syringes and a female-to-female Luer-Lock connector with a 1.6-mm internal diameter, until a homogeneous, milky-white emulsion formed (Supplementary Video 3). This emulsion was centrifuged at 1,600 g for 3 min. After removal of the inferior liquid and superior oil layers, the stromal vascular fraction gel (SVF-gel) was immediately prepared for subsequent applications (Supplementary Video 4).

**Figure 1:**
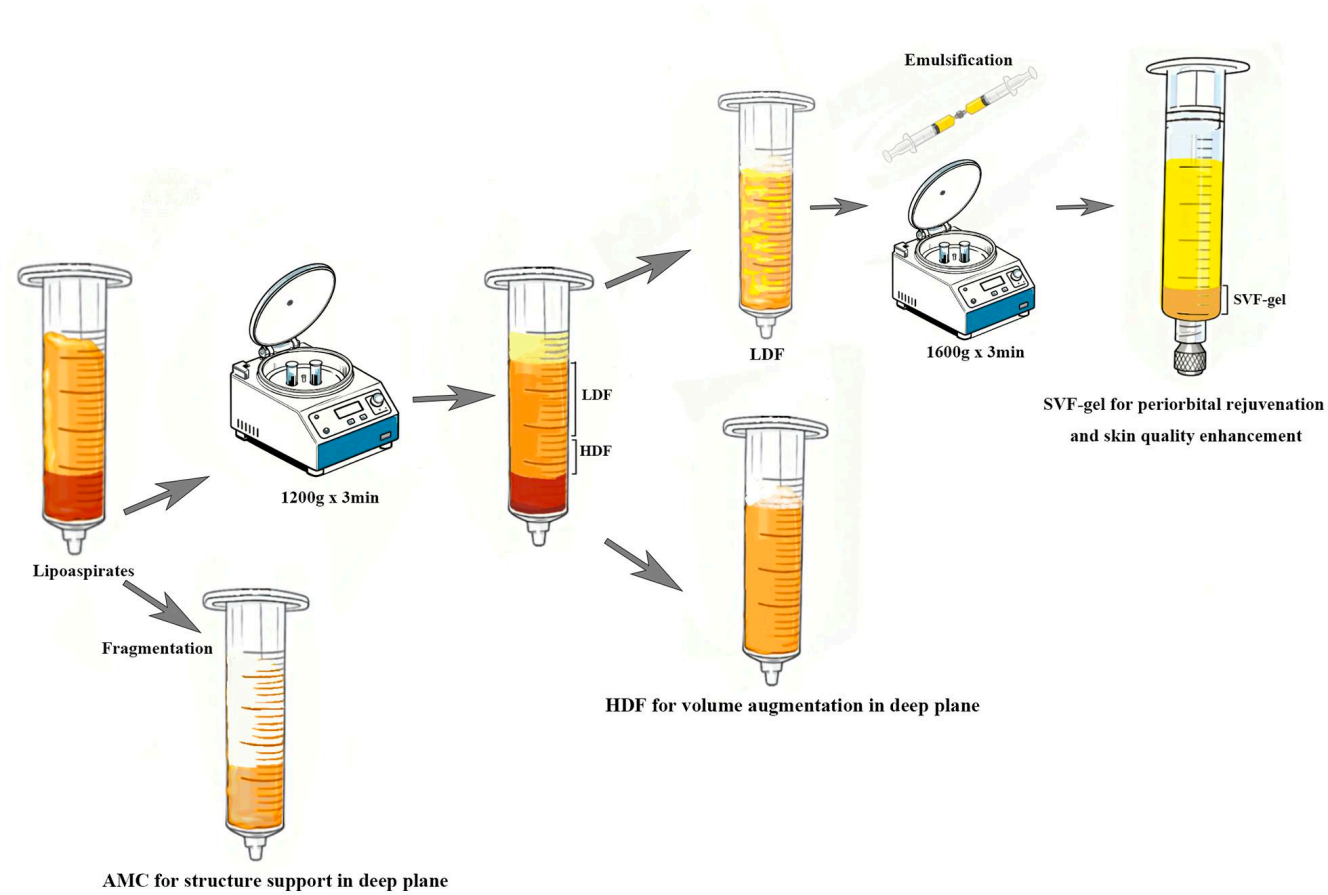
Schematic illustration of processing and injection protocols of adipose products: high-density fat (HDF), adipose matrix complex (AMC), and stromal vascular fraction gel (SVF-gel).

Local infiltration anesthesia was applied to the recipient area. Fat injection across all facial regions was administered using a 1 mL syringe with a 1.2 mm injection blunt cannula (Supplementary Video 5). The fat grafts were delivered under low injection pressure during slow, retrograde withdrawal of the cannula to ensure even distribution and minimize tissue trauma. Injection filling is performed separately at both the superficial and deep layers for each area. The superficial layer is uniformly injected with SVF-gel, while the choice of injection material for the deep layer varies depending on the specific region. Areas requiring certain structural support, such as the nasal base and chin, are treated with AMC, whereas other facial regions are typically filled with high-density adipose tissue. Injection depths were tailored to anatomical regions: the temporal area received subcutaneous and deep temporal fascial injections; the frontal region was treated in the subcutaneous and subgaleal planes; the cheek was addressed in sub superficial musculoaponeurotic system and subcutaneous layers. The nasolabial fold, nasojugal fold and the chin underwent both deep and superficial injection. The procedure involves first injecting the deep layer to achieve adequate fullness in the depressed areas, followed by superficial injection of SVF-gel into the subcutaneous fat layer to achieve skin tightening. Therefore, the volume and ratio of the materials used for deep and superficial injections are primarily determined by the degree of depression and the skin tension. The process and injection of adipose tissue is shown in Figure 1.

Photographs were taken preoperatively, postoperatively, and at all follow-up visits. At the 6-month follow-up, patients self-reported satisfaction using a Visual Analog scale (VAS) ranging from 0 (extremely unsatisfied) to 10 (very satisfied). Concurrently, a non-surgeon investigator assessed aesthetic improvement using the Global Aesthetic Improvement Scale (GAIS), scoring from −1 (worsened) to 3 (greatly improved).

## Results

A total of 105 patients (85 female, 20 male) underwent facial fat grafting. All patients provided written informed consent. The mean age was 35.5 years (range: 26–50 years), with a mean BMI of 23.2 ± 1.9 kg/m^2^. The mean follow-up period was 10 months (range: 6–15 months). Two patients developed significant erythema and elevated skin temperature within 1 week postoperatively, both of whom responded well to antibiotic therapy. Four patients reported bilateral asymmetry, while twelve expressed a desire for enhanced fullness. All sixteen patients subsequently underwent secondary filler augmentation at the 3-month follow-up. Patients undergoing secondary procedures and patients receiving a single procedure showed no statistically significant differences in gender, age, and BMI (*p* < 0.05).

Fat grafting was performed at seven anatomical sites, with each patient receiving treatment at one or multiple locations. Details regarding the injection location, volume and layers are summarized in Supplementary Table 1. Based on the Visual Analog Scale (VAS, 0–10), patient-reported satisfaction scores were 5.26 ± 1.84 preoperatively and 8.01 ± 1.09 postoperatively (Supplementary Table 2). Observer assessments using the Global Aesthetic Improvement Scale (GAIS, −1 to 3) yielded a postoperative score of 1.88 ± 0.65. The agreement between the two independent assessors was analyzed using Cohen's kappa statistic. The analysis demonstrated excellent inter-rater reliability [*κ* = 0.796, 95% CI (0.71, 0.86), *p* *<* 0.001].

### Case reports

#### Case 1

**Figure 2:**
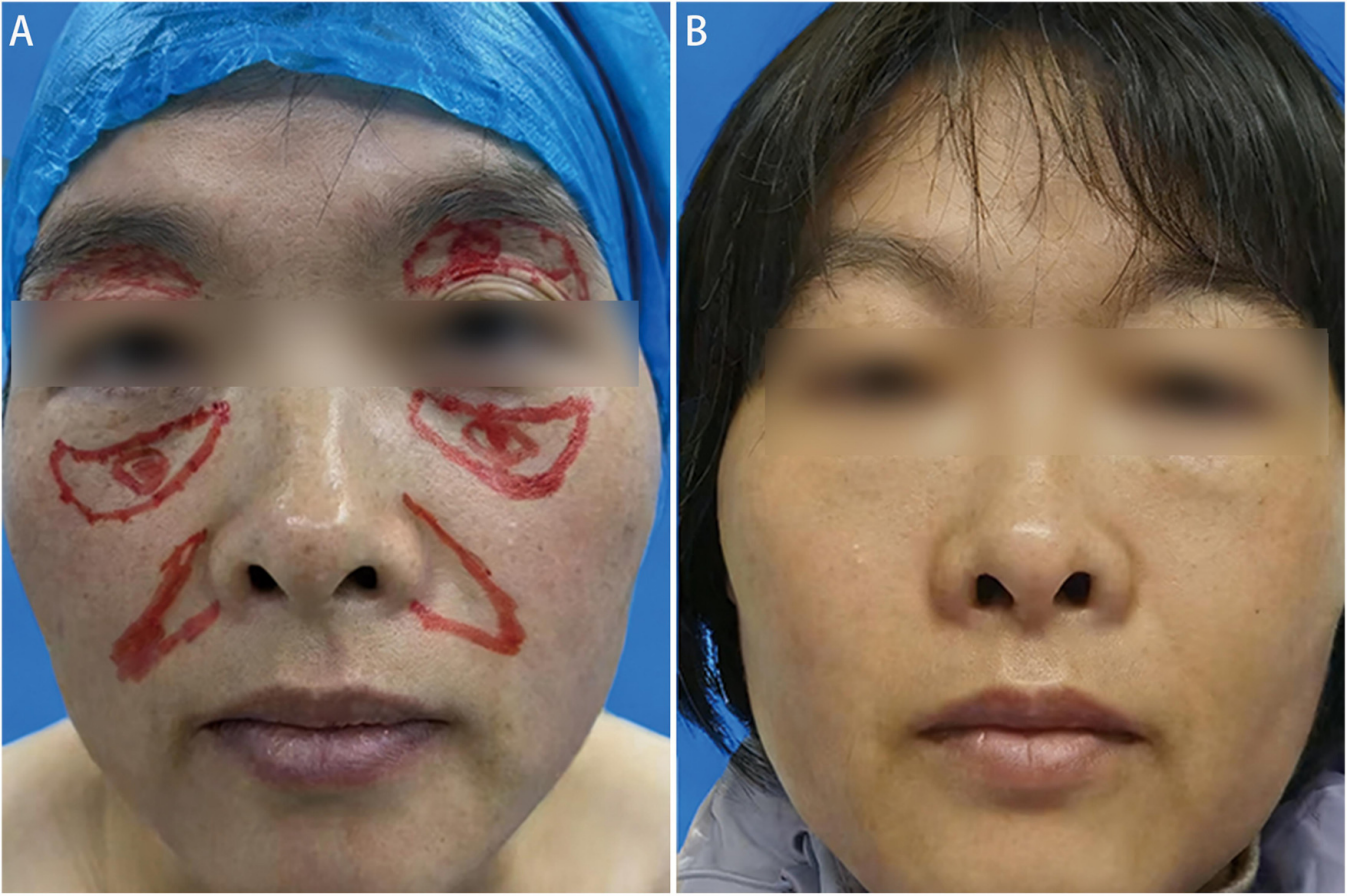
Multilayer facial augmentation in a 41-year-old female with hollowing in upper eyelid, nasojugal and nasolabial groove. SVF-gel was placed in the retro-orbicularis oculi fat, while high-density fat and adipose matrix component injected deeply, with SVF-gel distribution superficially in other regions. (**A**) Preoperative view. (**B**) Outcome at 8 months following injection.

A 35-year-old female patient presented with hollowing of the nasojugal and nasolabial grooves. HDF were injected into the supraperiosteal plane of nasojugal grooves, and AMC was injected in the supraperiosteal plane of nasolabial groove. SVF-gel was used in the superficial subcutaneous layer of both areas. The patient was followed for 1 year and reported satisfaction with the volumetric improvement. Preoperative and 1-year postoperative outcomes are illustrated in Figure 2.

#### Case 2

**Figure 3:**
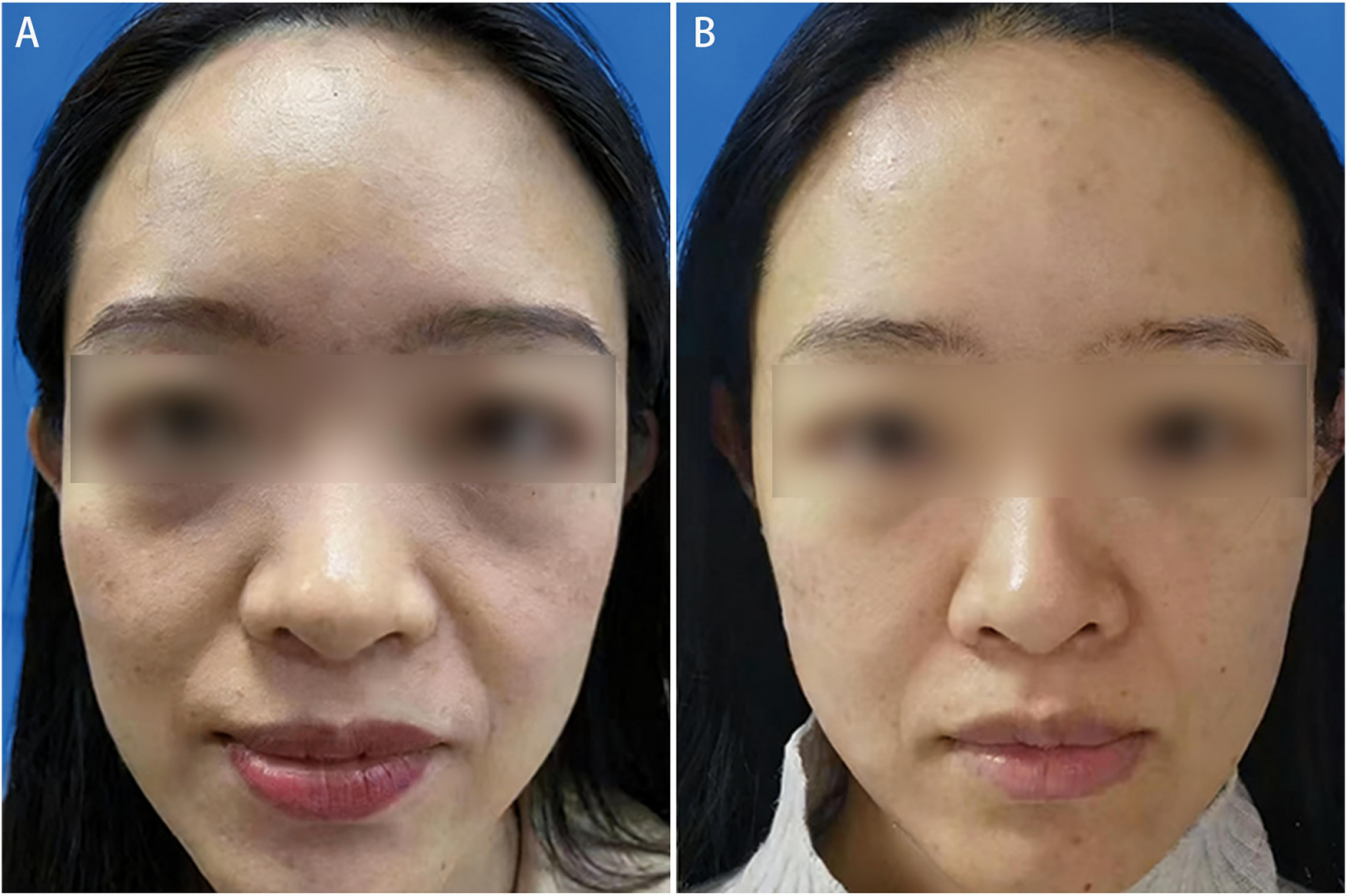
Correction of nasojugal and nasolabial grooves after multilayer augmentation in a 35-year-old female patient. High-density fat and adipose matrix component were placed in deep planes for structural support, while stromal vascular fraction gel was distributed superficially. (**A**) Preoperative appearance. (**B**) Postoperative view at 1 year.

A 41-year-old female patient presented with upper eyelid hollowing, with depression in the nasojugal and nasolabial grooves. SVF-gel was injected into the retro-orbicularis oculi fat (ROOF) pad. For nasojugal and nasolabial groove augmentation, HDF and AMC were separately injected into the deep plane, along with SVF-gel in the superficial subcutaneous plane. The patient expressed satisfaction with the procedural outcomes at the 8-month postoperatively. Preoperative and 8-month postoperative results are shown in Figure 3.

## Discussion

Facial aging is a complex, multi-layered phenomenon driven by interdependent changes across all tissue planes (15). Its manifestations extend beyond superficial cutaneous signs, such as wrinkles, loss of elasticity, and pigmentary changes. To include profound volumetric depletion and structural recontouring of the deep fat compartments and underlying skeleton (3, 16). His synergistic decline in both surface and structural support fundamentally defines the aged facial appearance. In 1990s, Coleman first established a standard protocol for facial fat grafting (17). Since then, Autologous fat grafting has emerged as a safe, abundant, easily available, and biocompatible technique for facial atrophy with predictable results. Over decades, refinements in harvesting and processing methodologies, leading to the deliberate preparation of adipose grafts with varied cellular profiles and tissue parcel sizes, each aiming to optimize specific aspects of restoration (18, 19).

Adipose tissue is typically categorized into three main types: macrofat, microfat, and nanofat (12). Macrofat exhibits adipose parcel diameters of 2–2.5 mm and is usually harvested using a 3-mm cannula. In comparison, microfat consists of parcels approximately 1 mm in size, obtained with cannulas measuring 2.1–2.4 mm in diameter (20). Macrofat and microfat, composed predominantly of mature adipocytes, are mainly applied for the correction of soft tissue defects and the restoration of volume (21). In contrast, nanofat is produced through mechanical emulsification, which yields a product rich in stromal vascular fraction (SVF), extracellular matrix, and growth factors, but with minimal viable adipocytes. The SVF-rich composition is reported to promote tissue regeneration and improve skin quality by enhancing elasticity and reducing hyperpigmentation (22). During the preparation of nanofat, centrifugation and emulsification may induce mechanical trauma to the adipose tissue, potentially compromising adipocyte viability, which was also a concern noted by some investigators. While quantitative data detailing the extent of this mechanical damage remain unreported, the processed nanofat achieves a substantially higher cellular density. This enhanced density and purity enables effective volumetric augmentation, clinical outcomes corroborated by its established long-term safety and efficacy profile in practice (21, 22). Thus, various processed adipose products exhibit unique biological compositions and functional properties. Consequently, facial rejuvenation via fat grafting targets both volumetric restoration and the promotion of tissue repair and regeneration.

Based on established protocols, centrifuged adipose tissue stratifies into high-density fat (HDF) and low-density fat (LDF) (23) HDF, collected from the lower half portion of centrifuged Coleman fat, is enriched with viable adipocytes, stromal vascular fraction (SVF) cells, and extracellular matrix (ECM) relative to LDF (24). In contrast, LDF constitutes the upper layer and contains a substantial oil fraction, which has been linked to a higher risk of postoperative complications such as oil-cyst formation (25). Consequently, HDF exhibits superior graft retention and a lower complication profile compared to both conventional Coleman fat and LDF (26). This enhanced structural and biological integrity supports the preferential use of HDF in deep structural augmentation of the face. During operation, the injection plane for HDF was usually the deep fascial plane in which advancement through and injection met with moderate resistance to make sure at the accurate plane.

Adipose tissue, while effective for volumetric restoration, offers limited mechanical support due to its inherent low stiffness (27). The stiffness of adipose tissue depends mostly on proportion of ECM, which includes different types of collagen (28). Therefore, the demand for identifying adipose products with enhanced mechanical properties is critical for achieving optimal structural support in facial augmentation. The adipose matrix complex (AMC), isolated from high-density fat (HDF) via filtration, represents such an advanced product (29). By retaining abundant collagen while reducing the adipocyte fraction, AMC exhibits superior stiffness and rigidity. These enhanced mechanical properties, coupled with its reported higher retention rate compared to conventional Coleman fat, make AMC particularly suitable for periosteal injection in areas demanding robust structural reinforcement, such as the chin, alar base, eyebrow arch, zygomatic arch, and nasal dorsum. For these regions, the deep injection plane was determined mainly based on tactile feedback during needle advancement. A distinct, firm endpoint was encountered upon contacting the periosteum in deep structural areas.

Periorbital hollowing is increasingly recognized as a significant feature of facial aging. Nevertheless, the application of HDF or AMC for volume augmentation in this region is suboptimal compared to other facial areas, primarily due to their large particle size. SVF-gel, with its uniform emulsified consistency and smooth texture, appears suitable to minimize nodule formation and surface irregularity. SVF-gel contains abundant ADSCs and other SVF cells while possessing reduced oil droplet content, contributing to higher retention rates compared with conventional fat grafts (30). The regenerative capacity of ADSCs, as primary agents of collagen deposition and neovascularization, facilitates wound healing and underlies the skin texture refinement achieved with SVF-gel grafting. This treatment modality has been validated in clinical settings for tear trough correction and lip augmentation (8, 31). Based on our clinical experience, SVF-gel serves two essential functions in facial rejuvenation: first, as a volumetric filler for supraorbital and infraorbital hollowing; second, as a complementary graft injected superficially alongside conventional fat transplantation in other facial regions to enhance skin quality and reduce rhytids.

In addition, the injection volumes and layering strategies were determined intraoperatively through an individualized, anatomy-based approach. Injection volumes were individualized based on the specific volumetric deficit, the patient's unique facial morphology including skeletal support and gender-specific ideals, and the preoperative aesthetic goal. In our clinical series, female patients predominantly seek a fuller, more voluminous appearance, whereas male patients typically request correction of volume deficits to restore a natural contour. Thus, the injection endpoint is individually tailored. For patients desiring subtle improvement, we aim for moderate correction where the overlying skin retains natural skin resilience. For those pursuing maximal fullness, we inject until the recipient site approached its volumetric capacity achieving mild skin tension. Therefore, in our multilayer facial injection strategies, the superficial layer is injected with SVF-gel for enhancement of skin quality and deep layer is injected with high-density adipose tissue or AMC depending on the specific region for structural volumetric augmentation and certain structural support. The superficial subcutaneous plane was identified by palpable needle movement directly beneath the dermis.

Through patient-reported satisfaction scores and observer objective assessment, the strategy of multilayer facial augmentation with processed adipose products was an effective approach for facial rejuvenation. However, there exist several limitations in this study. The absence of quantitative volumetric analysis limits the precise determination of fat graft survival rate and the efficacy of facial depression correction. Furthermore, lack of a comparative analysis between different injection techniques hinders a systematic evaluation of the relative advantages and limitations of various surgical approaches and distinct adipose-derived products. Additionally, given the inherent variability in fat graft survival, quantitative analyses were conducted at the 6-month postoperative interval in this study. Longer-term follow-up is warranted to further evaluate graft survival and structural changes. The retrospective nature of the study also limits the ability to control for confounding variables that might have influenced the outcomes.

## Conclusion

In conclusion, this study demonstrates that the strategic application of differentially processed adipose products, according to specific anatomical requirements and defect characteristics, enables comprehensive facial rejuvenation. Patient-reported satisfaction scores and observer objective assessment have verified the strategy of multilayer facial augmentation with processed adipose products was an effective approach for facial rejuvenation in which structural augmentation with HDF and AMC in deep layers to superficial refinement using SVF-gel. The multilayer approach effectively restores volume, provides structural support, and enhances skin quality. Future studies incorporating objective measurements, controlled comparisons, and extended follow-up will further refine these techniques and establish evidence-based guidelines for optimal product selection and application in facial contour restoration.

## Data Availability

The original contributions presented in the study are included in the article/**Supplementary Material**, further inquiries can be directed to the corresponding author.
