# Supplementary material for: Strategic application of multilayer fat grafting in facial rejuvenation: a retrospective study
Source: Front Surg. 2026 Apr 1;13:1744865. doi: 10.3389/fsurg.2026.1744865 (PMC13078975; doi:10.3389/fsurg.2026.1744865)
Supplement: Supplementary file 1 [file Table1.docx]

**Supplementary Table 1 Application of multilayer facial fat grafting.**

| **location** | **Value** | **Injection layer** | **Type of fat product** | **Fat graft volume per side (mL)** |
| --- | --- | --- | --- | --- |
| Forehead | 19 | subgaleal | HDF | 7.6 ± 1.2 |
|  |  | superficial subcutaneous | SVF-gel | 2.6 ± 0.4 |
| Temple | 83 | deep temporal fascial | HDF | 9.1 ± 1.3 |
|  |  | superficial subcutaneous | SVF-gel | 3.1 ± 0.5 |
| Cheek | 36 | sub-SMAS | HDF | 6.8 ± 1.1 |
|  |  | superficial subcutaneous | SVF-gel | 3.5 ± 0.5 |
| Superior sulcus | 40 | ROOF | SVF-gel | 1.6 ± 0.3 |
| Nasojugal groove | 87 | supraperiosteal plane | HDF | 1.2 ± 0.1 |
|  |  | superficial subcutaneous layer | SVF-gel | 0.6 ± 0.2 |
| Nasolabial groove | 93 | supraperiosteal plane | AMC | 1.9 ± 0.3 |
|  |  | superficial subcutaneous layer | SVF-gel | 1.8 ± 0.4 |
| Chin | 46 | supraperiosteal plane | AMC | 2.1 ± 0.3 |
|  |  | superficial subcutaneous layer | SVF-gel | 2.0 ± 0.3 |

HDF, high-density fat; AMC, adipose matrix complex; SVF-gel, stromal vascular fraction gel; ROOF, retro-orbicularis oculi fat; SMAS, superficial musculoaponeurotic system
