# Supplementary material for: Strategic application of multilayer fat grafting in facial rejuvenation: a retrospective study
Source: Front Surg. 2026 Apr 1;13:1744865. doi: 10.3389/fsurg.2026.1744865 (PMC13078975; doi:10.3389/fsurg.2026.1744865)
Supplement: Supplementary file 2 [file Table2.docx]

**Supplementary Table 2 Patient-reported outcome assessment.**

| Question  (0=extremely dissatisfied.  10= totally satisfied) | Mean score (standard deviation) | | *p* |
| --- | --- | --- | --- |
|  | Preoperative | Postoperative |  |
| Q1: Satisfaction with facial appearance overall | 6.07 (1.61) | 7.91 (0.99) | <0.001 |
| Q2: Satisfaction with depressed region | 3.78 (1.33) | 8.33 (0.94) | <0.001 |
| Q3: Wrinkles and texture of depressed region | 4.47 (1.82) | 7.54 (1.28) | <0.001 |
| Q4: Appearance-related self-confidence | 5.84 (1.56) | 8.09 (1.05) | <0.001 |
| Q5: Quality of social life | 6.13 (1.53) | 8.15 (0.86) | <0.001 |
| Overall satisfaction | 5.26 (1.84) | 8.01 (1.09) | <0.001 |
